# A comprehensive evaluation of pregnancy and newborn outcomes in Syrian refugees in Turkey

**DOI:** 10.1186/s12884-023-06168-2

**Published:** 2024-01-02

**Authors:** Aylin Önder Dirican, Dilay Gök Korucu

**Affiliations:** 1 Department of Gynecology and Obstetrics, Başkent University Konya Practice and Research Hospital, Konya, Turkey; 2 Department of Gynecology and Obstetrics, Konya City Hospital, Konya, Turkey

**Keywords:** Syrian refugees, Obstetric outcome, Maternal complication, Neonatal outcome Vitamin D

## Abstract

**Background:**

The research was conducted to evaluate the birth and newborn outcomes of Syrian immigrant women according to maternal age groups and Vitamin D use.

**Methods:**

It was conducted retrospectively using the birth records of 2,866 Syrian migrant women, who had given birth at a tertiary center between January 2016 and May 2020. Demographic features, obstetric and neonatal outcomes were analyzed according to age groups and Vitamin D use.

**Results:**

The mean age of the patients included in the study was 26.22 ± 5.90, the mean gestational age at birth was 38.06 ± 2.1 weeks, and the mean newborn birth weight was 3.151 g. The mean hemoglobin value of the patients was 11.55 ± 1.54. While most of the patients were taking iron supplements (80.59%), Vitamin D (Vit D) supplement intake was 38.31%. The mean number of antenatal follow-ups was 3.40 ± 1.65. While the most common delivery method was normal vaginal delivery (61.93%), cesarean section rates were found to be 38.07%. The need for blood transfusion was significantly lower in the group that had received Vitamin D than that in the group that had not received it (2.00% vs. 8.94% p < 0.001). The rate of preterm birth was found to be 5.74% in the group that had received Vitamin D and 9.28% in the group that had not received it, which was significantly higher (p < 0.001).

**Conclusions:**

We have seen that maternal and fetal outcomes can be improved with hospital follow-up and adequate vitamin supplements in refugee pregnant women.

## Background

As a result of the war that started in Syria about 10 years ago, Syrians had to migrate to neighboring countries such as Jordan, Lebanon and Turkey [1]. Turkey continues to host the largest number of refugees in the world [2]. In September 2020, the total number of Syrian refugees in Turkey was about 3.6 million (63.4% of all Syrian refugees). While 46% of registered Syrian migrants are women, the number of women aged 15–49 is approximately 800,000. Konya is one of the cities to which a significant number of refugees migrated. In Konya, where 5.2% of the population are Syrian immigrants, there are approximately 116,000 refugees, 46% of whom are women [3, 4]. As recipients of “temporary accommodation status” Syrians are able to benefit from basic health and social services. Pregnancy health services, including interpretation services, are provided free of charge to Syrian immigrants in state hospitals in Turkey [4].

However, having to live in a different geography away from their homes, communication problems and low income levels can naturally affect individuals’ access to health services. This situation may also have adversely affected reproductive health and prenatal care [1, 5]. Many studies report poor reproductive health outcomes such as preterm birth, increased incidence of cesarean section (CS), intrapartum hemorrhage, gestational diabetes, and puerperal infections among refugee women in different countries. In addition, infants of refugee mothers have been shown to have adverse neonatal outcomes such as low birth weight (LBW), long hospital stays, low appearance of the newborn at the 1st and 5th minutes, Pulse, Grimace Response, Activity, and Respiration (APGAR) scores [2, 6, 7].

Maternal and child health in a country can be affected by many structural and social factors. Therefore, outcomes related to maternal and child health vary from country to country. Pre-pregnancy care and antenatal follow-up are very important for predicting and managing many complications that may occur during delivery. At the same time, inadequate pre-pregnancy care, malnutrition and vitamin intake can lead to poor pregnancy outcomes. The World Health Organisation (WHO) recommends eight antenatal care visits during pregnancy, the first within the first 12 weeks [8]. According to the “Antenatal Care Management Guidelines”, the Republic of Türkiye Ministry of Health recommends at least four follow-ups (the first during the first 12 weeks of pregnancy, then between 18 and 24, 28–32, and 36–38 weeks) in normal pregnancies. Again, according to this guide, education and information about prenatal fetal screenings, necessary laboratory tests as well as iron and Vitamin D supplements are essential. In accordance with the same guideline, the recommendation for Vitamin D supplementation in pregnant women is to commence with 1200 IU once a day starting from the 12th week of pregnancy and to continue throughout the entire pregnancy and for the first 6 months after birth [9].

The WHO defines adolescence as occurring between the ages of 10–19. The adolescent pregnancy rate, which has become a global problem, is approximately 11%, and 90% of these pregnancies occur in low- or middle-income countries [10]. Adolescence is a special period in many ways, and growth and development in this period can affect the long-term general health of individuals and even the children they will have in later life. Increasing adolescent pregnancy rates in many countries continue to endanger the growth and development of subsequent generations and adolescent girls [11]. Pregnant adolescents face various problems such as changes in nutrition and social life, language barriers and limited access to hospitals, especially when civil wars force them to migrate from their countries. Many studies have shown that conditions such as increased cesarean delivery rates, preterm birth, pregnancy-induced hypertension (PIH), anemia, fetal distress, oligohydramnios and gestational diabetes are more common in adolescent pregnancies [12, 13].

Many medical societies and the WHO do not recommend the routine use of dietary supplements for all pregnant women. However, a balanced diet that can provide adequate minerals and vitamins before and during pregnancy is among the current recommendations. As migrant pregnant women are particularly at risk of malnutrition, it is important that they have access to necessary vitamin and mineral supplements. Iron deficiency anemia is the most common nutritional deficiency among pregnant women. Vitamin D deficiency, especially during pregnancy preeclampsia, is releated with gestational diabetes mellitus, cesarean section and it has been shown to increase bacterial vaginosis [14]. Vitamin D has effects such as modulation of immunity, regulation of cellular growth and differentiation, and the induction of erythropoiesis in bone marrow cells. In addition, studies show that Vitamin D supplements improve hemoglobin and ferritin levels [15, 16].

Since 2011, Turkey has continued to accept Syrian refugees. Although health services are available for Syrian immigrants in Turkiye, their poor socioeconomic status, language and cultural differences can negatively affect their reproductive health. The aim of this study is to evaluate the obstetric and perinatal outcomes in a tertiary center with a large Syrian refugee pregnant population. In addition, evaluating the effects of vitamin supplements on adolescent pregnancy and outcomes are a secondary gain.

## Methods

### Study design and setting

This study was planned as a retrospective descriptive study in Konya Training and Research Hospital between 2016 and 2020. Birth records of a total of 2985 Syrian refugee women reviewed retrospectively. Our hospital is a tertiary center that provides the same service to all pregnant women, whether they are refugees or not, and has a high annual birth rate. The study was approved by the Konya Karatay University Clinical Research Ethics Committee (approval number 2023/028).

### Participants and data collecting

The participants of the study were Syrian women, who had given birth between 2016 and 2020. The number of visits, examination and laboratory findings at each visit, birth and surgery reports, newborn reports, and prescription charts were examined and recorded for each of the women. A total of 2,866 Syrian refugees were included in the study, excluding those who had given birth before 24 weeks of age, those who had serious systemic comorbidities, had had multiple pregnancies, and those in respect of whom there were missing data.

### Variables

Maternal age, income level, education level, number of births, number of miscarriages, mode of delivery, number of antenatal follow-ups, chronic diseases and pregnancy diseases were screened. The use of folic acid, Vitamin D and iron supplements from the prescriptions received from the e-prescription system during the pregnancy of the women was recorded. Gestational age at birth, mode of delivery, APGAR scores, birth weight and, if necessary, hospitalization to the neonatal intensive care unit (NICU) were recorded. In addition, the mother’s need for blood transfusion and hospital stay were recorded. Gestational age was calculated according to the last menstrual period (LMP), if the LMP is unknown, according to the first-tremester ultrasonography head-rump length (CRL) measurement, and if there is no follow-up, according to the last ultrasonography measurement. All variables were compared according to age groups (under 20 years of age, 20–35 years old, and over 35 years old), and Vitamin D use.

### Definitions

According to the International Health Classification System, preterm birth was defined as birth before 37 weeks of gestation following the last menstrual period [10]. Anemia is defined as low hemoglobin concentrations relative to trimester-specific cut-off values. Anemia is accepted if Hb is < 11.0 g/dl in the third trimester [17]. In our study, hemoglobin levels were evaluated at admission for delivery [third trimester] and Hb < 11.0 g/dl was accepted as anemia. In our hospital, all pregnant women are provided with vitamin supplements, specifically 1200 IU of Vitamin D per day., starting from the 12th week of pregnancy, in line with the directives of the Ministry of Health of the Republic of Turkey [9]. Preterm premature rupture of membranes (PPROM) was defined as rupture of membranes before 37 weeks of gestation [18]. Pregnant women, who accepted to have an oral glucose tolerance test (OGTT) were screened for one- or two-stage gestational diabetes (GDM) between 24 and 28 weeks. The diagnosis of GDM was made according to the two-stage (50-100 g) or single-stage (75 g) OGTT criteria defined by the 2018 American Society of Gynecology and Obstetrics [19]. Hypertensive disorder of pregnancy (GHT) was defined as the coexistence of new-onset hypertension (≥ 140/90 mm Hg) and/or proteinuria (≥ 300 mg/24 hours) after 20 weeks of gestation in a previously normotensive woman [20]. Postpartum hemorrhage includes all bleeding due to postpartum uterine atone, placental adhesion anomalies, placental abruption, and severe vaginal lacerations. According to the birth weight of the newborns, macrosomia ≥ 4000 g, normal birth weight > 2500 g, babies born between 2500 g and 1500 g were defined as low birth weight (LBW), babies born between < 1500 gr as very low birth weight (VLBW) [21].

### Statistical analysis

Numerical variables, mean and standard deviation, and categorical variables, frequency and percentage were shown with descriptive statistics. Chi-square and Fisher precision tests were used in the analysis of categorical variables. In the analysis of numerical variables, the t test, which is one of the ANOVA tests, was used. The Tukey test was used for pairwise comparisons. Statistical analysis was performed using the R 4.2.2 (R CoreTeam 2023) program. A *p*-value < 0.05 was considered significant.

## Results

A total of 2.866 Syrian refugee women, who gave birth were included in the study. All demographic and clinical parameters are summarized in Table 1. Most of the Syrian women [79.20%] were between the ages of 20–34 and the mean age was 26.22 ± 5.90 years. The percentage of women in adolescence [under 20 years old] was 10.82%. The mean last pregnancy interval was 22.08 ± 14.75 months in women with a mean gravity of 3.91 ± 1.90, and parity 2.57 ± 1.59. Mean gestational age at birth was 38.6 ± 2.1 weeks and mean birth weight was 3.147.25 ± 529.48 g. The mean number of antenatal follow-ups of Syrian women during their pregnancies was 3.40 ± 1.65, and the mean Hb values ​​at the time of delivery were 11.55 ± 1.54. While most of the patients (80.57%) were taking iron supplements, the rate of Vitamin D supplement intake was 38.31%. While the most common type of delivery was normal vaginal delivery [61.93%], the primary cesarean section rate was 5.69%. The indications for cesarean section were previous cesarean delivery (84.60%), cephalopelvic discordance (CPD 7.24%) and breech presentation (5.13%), respectively. The most common pregnancy complications were postpartum hemorrhage (2.51%), hypertensive disorder of pregnancy (1.22%), GDM (1.08%), and PPROM (0.66%). The rate of delivery at < 37 weeks (preterm labor) was 7.92%. In addition, 6.28% of women required blood transfusions, while 5.69% had a consistent cesarean hysterectomy.

**Table 1:** Maternal and fetal demographic and clinical outcomes

Variable		n = 2,866
Age (year) *(Mean ± SD)*		26.22 ± 5.90
Age groups (years), *n (%)*		310.00 (10.82%)
	< 20	2,270.00 (79.20%)
	20–34	286.00 (9.98%)
	> 35	
Education Level, *n (%)*		
	Uneducated	576.00 (20.10%)
	Primary Education	1,752.00 (61.13%)
	High School	576.00 (20.10%)
Economic Status, *n (%)*		
	High	388.00 (13.54%)
	Intermediate	1,213.00 (42.34%)
	Lower	1,264.00 (44.12%)
Duration of Marriage Year *(Mean ± SD)*		7.53 ± 4.87
	Gravidity	3.91 ± 1.90
	Parity	2.57 ± 1.59
	Live Births	2.53 ± 1.56
	Abortus	0.35 ± 0.87
Last Pregnancy Interval, Month *(Mean ± SD)*		22.08 ± 14.75
Gestational Age at Birth, Week *(Mean ± SD)*		38.6 ± 2.1
Hb (g/dl) (Mean ± SD)		11.55 ± 1.54
Hematocrit (%) *(Mean ± SD)*		35.28 ± 4.16
Number of antenatal follow-ups *(Mean ± SD)*		3.40 ± 1.65
Iron (Fe) Supplement, *n (%)*		2,309.00 (80.57%)
Vitamin D Supplement, *n (%)*		1,098.00 (38.31%)
**Obstetric results** *n (%)*		
Delivery Type		
	Vaginal Delivery	1,775.00 (61.93%)
	Cesarean Delivery	1,091.00 (38.07%)
	Cesarean Hysterectomy	14.00 (0.49%)
	Primary C-section Prevalence	163.00 (5.69%)
Caserean Section Indications		
	Previous C-section	923.00 (84.60%)
	CPD	79.00 (7.24%)
	Breech presentation	56.00 (5.13%)
	Other	33.00 (3.02%)
Hypertensive disorders of pregnancy		35.00 (1.22%)
GDM		31.00 (1.08%)
PPROM		19.00 (0.66%)
Postpartum hemorrhage		72.00 (2.51%)
Blood transfusion		180.00 (6.28%)
Length of hospitalization, day, *(Mean ± SD)*		1.46 ± 1.06
**Neonatal results**		
	Preterm Labor (< 37 weeks), *n (%)*	227.00 (7.92%)
	Birth weight (g) *(Mean ± SD)*	3,151.14 ± 519.51
APGAR Scors *(Mean ± SD)*		
	1. Minutes	7.06 ± 0.69
	5. Minutes	8.92 ± 0.59
NICU admission (*n*, %)		136.00 (4.75%)
Stillbirth (*n*, %)		18.00 (0.63%)
Congenital anomaly (*n*, %)		89.00 (3.08%)
Macrosomia (≥ 4000 g) (*n*, %)		81.00 (2.83%)
Gender (*n*, %)		
	Male	1,460.00 (50.94%)
	Female	1,404.00 (48.99%)

One-way ANOVA; Pearson’s Chi-squared test; Kruskal-Wallis rank sum test; Fisher’s exact test

SD: Standard deviation, n: number, %: percent, CPD: cephalopelvic dispropotion, GDM: Gestational diabetes mellitus, PPROM: preterm premature rupture of membrane, NICU: Neonatal Intensive Care Unit

While 98% of the newborns were of normal birth weight (> 2500 g), 2.89% were macrosomic (≥ 4000 g). The mean 1st and 5th minute APGAR scores of newborns were 7.06 ± 0.69 and 8.92 ± 0.59, respectively, and 4.75% were admitted to the NICU. While the stillbirth rate was 0.63%, fetal congenital anomaly rate was 3.08% (Table 1).

Variables according to maternal age groups are compared in Table 2. Education level and economic status in the adolescent (< 20 years) period were significantly lower (p < 0.001) compared to other age groups (20–34 and > 35 years). The last pregnancy interval of patients under 20 years of age was 10.48 ± 5.95 months, which was significantly lower than other age groups [p < 0.001]. Gestational age at birth [weeks] was similar in all age groups (p = 0.9). Birth weights (grams) were 3.097.24 ± 495.26 in the < 20 age group, 3.147.19 ± 513.11 in the 20–35 age group, and 3.240.89 ± 582.84 in the > 35 age group and increased significantly with maternal age (p = 0.007). Consistent with this result, the rate of macrosomic infants (> 4000 g) was 0.32%, 2.69% and 6.64% in the < 20, 20–35 and > 35 age groups, respectively, and increased significantly (p < 0.001) with maternal age. Although the hemogram [Hb] and hematocrit (Hct) values ​​increased with increasing maternal age, this increase did not reach significance (p = 0.052, and p = 0.059, respectively). Iron and Vitamin D supplement intake was lowest in the < 20 age group, and iron supplement intake in particular was significantly higher in the 20–35 (80.88%) and > 35 (83.92%) age groups (p = 0.019). While cesarean section was significantly higher (p < 0.001) in the > 35 age group (51.40%), vaginal delivery was significantly higher (p < 0.001) in the < 20 age group (84.19%). In addition, primary cesarean section rates increased significantly (p < 0.001) with increasing maternal age. When the age groups were compared in terms of pregnancy complications, the rates of preterm birth and PPROM were similar in all age groups (p > 0.9, and p = 0.06, respectively), while the rates of hypertensive disorder of pregnancy, GDM, postpartum hemorrhage and blood transfusion were higher in the > 35 age group. When the age groups were compared in terms of pregnancy complications, while the rates of preterm birth and PPROM were similar in all age groups (p > 0.9, and p = 0.06, respectively), the rates of hypertensive disorder of pregnancy, GDM, postpartum hemorrhage and blood transfusion were significantly higher than those in the > 35 age group (p < 0.001, p = 0.015). In the < 20 age group, the 1st minute mean APGAR score (6.91 ± 0.69) was significantly lower than that in the other age groups (p < 0.001). While neonatal NICU admission rates were higher in the < 20 age group (7.1%) than in the other two groups (20–35, 4.49% and > 35, 4.20%), there was no significant difference (p = 0.12).

**Table 2:** Comparison of variables in terms of maternal age groups

Variables		< 20	20–34	> 35	p
		n = 310	n = 2,273	n = 287	
Age		17.92 ± 1.25	25.89 ± 4.08	37.80 ± 2.38	**< 0.001**
Education Level, *n (%)*					**< 0.001**
	Uneducated	135.00 (43.55%)	326.00 (14.36%)	77.00 (26.92%)	
	Primary Education	169.00 (54.52%)	1,467.00 (64.63%)	116.00 (40.56%)	
	High School	6.00 (1.94%)	477.00 (21.01%)	93.00 (32.52%)	
Economic Status, *n (%)*					**< 0.001**
	High	6.00 (1.94%)	305.00 (13.44%)	77.00 (26.92%)	
	Intermediate	82.00 (26.45%)	1,039.00 (45.79%)	92.00 (32.17%)	
	Lower	222.00 (71.61%)	925.00 (40.77%)	117.00 (40.91%)	
Pregnancy history					
	Gravidity	2.52 ± 0.92	3.84 ± 1.61	6.04 ± 2.81	**< 0.001**
	Parity	1.37 ± 0.80	2.50 ± 1.38	4.34 ± 2.23	**< 0.001**
	Live Births	1.35 ± 0.80	2.47 ± 1.34	4.23 ± 2.20	**< 0.001**
	Abortus	0.15 ± 0.49	0.34 ± 0.81	0.69 ± 1.40	**< 0.001**
Last Pregnancy İnterval Month		10.48 ± 5.95	21.17 ± 12.04	41.87 ± 21.19	**< 0.001**
Duration of Marriage Year		3.24 ± 1.34	6.91 ± 3.46	17.11 ± 5.04	**< 0.001**
Number of antenatal Follow up		2.94 ± 1.69	3.44 ± 1.63	3.59 ± 1.62	**< 0.001**
Gestational Age at Birth		38.74 ± 2.13	38.69 ± 2.044	38.66 ± 1.98	0.9
Birth Weight		3,097.24 ± 495.26	3,147.19 ± 513.11	3,240.89 ± 582.84	**0.007**
Hb (g/dl)		11.42 ± 1.57	11.54 ± 1.53	11.73 ± 1.54	0.052
Htc (%)		34.89 ± 4.71	35.28 ± 4.04	35.70 ± 4.42	0.059
Iron (Fe) Supplement		233.00 (75.16%)	1,836.00 (80.88%)	240.00 (83.92%)	**0.019**
Vitamin D Supplement		103.00 (33.23%)	894.00 (39.38%)	101.00 (35.31%)	0.061
Delivery Type					**< 0.001**
	Cesarean Section	49.00 (15.81%)	895.00 (39.43%)	147.00 (51.40%)	
	Vaginal Delivery	261.00 (84.19%)	1,375.00 (60.57%)	139.00 (48.60%)	
Primary C-section Prevalence		6.00 (1.94%)	127.00 (5.59%)	30.00 (10.49%)	**< 0.001**
Preterm Labor		25.00 (8.06%)	179.00 (7.89%)	23.00 (8.04%)	0.6
	Birth weight 1500–2500 (g)	20.00 (11.83%)	126.00 (74.56%)	23.00 (13.61%)	**<0.001**
	Birth weight < 1500 (g)	6.00 (14.29%)	32.00 (76.19%)	4.00 (9.52%)	**< 0.001**
	Macrosomia (≥ 4000 g)	1.00 (0.32%)	61.00 (2.69%)	19.00 (6.64%)	**< 0.001**
Hypertensive disorders of pregnancy		0.00 (0.00%)	27.00 (1.19%)	8.00 (2.80%)	0.059
GDM		0.00 (0.00%)	19.00 (0.84%)	12.00 (4.20%)	**0.015**
Postpartum hemorrhage		3.00 (0.97%)	54.00 (2.38%)	15.00 (5.24%)	**< 0.001**
PPROM		2.00 (0.65%)	13.00 (0.57%)	4.00 (1.40%)	0.06
Blood transfusion		13.00 (4.19%)	139.00 (6.13%)	28.00 (9.79%)	**< 0.001**
Length of hospitalization, day		1.29 ± 0.90	1.45 ± 1.03	1.68 ± 1.39	**< 0.001**
APGAR Score					
	1.Minute	6.91 ± 0.69	7.08 ± 0.68	7.10 ± 0.73	**< 0.001**
	5. Minutes	8.87 ± 0.65	8.93 ± 0.59	8.92 ± 0.56	0.069
NICU admission		22.00 (7.10%)	102.00 (4.49%)	12.00 (4.20%)	0.12
Stillbirth		3.00 (0.97%)	14.00 (0.62%)	1.00 (0.35%)	> 0.9
Congenital fetal anomaly		12.00 (3.87%)	65.00 (2.86%)	12.00 (4.20%)	0.059

One-way ANOVA; Pearson’s Chi-squared test; Kruskal-Wallis rank sum test; Fisher’s exact test, SD: Standard deviation, n: number, %: percent, CPD: cephalopelvic dispropotion, GDM: Gestational diabetes mellitus, PPROM: preterm premature rupture of membrane, NICU: Neonatal Intensive Care Unit

The comparison of the variables according to the intake of Vitamin D supplements is given in Table 3. Number of antenatal visits (4.60 ± 1.21), gestational age at birth (38.9 ± 1.82), birth weight (3,195.46 ± 484.08), Hb (12.23 ± 1.29) and Hct (36.79 ± 3.98) values ​​were significantly higher in those who took Vitamin D supplements than in those who did not. (p < 0.001). The rates of low (LBW, 28.40%) and very low birth weight (VLBW, 2.91%) infants, who received Vitamin D were significantly lower than those who did not (LBW, 71.60%, VLBW, 5.08%) (p < 0.001). Preterm labor (5.74-9.28%), GDM (0.91-1.19%), postpartum hemorrhage (1.82-2.94%) and blood transfusion need (2.00-8.94%) were significantly lower in those who received Vitamin D than in those who did not (p < 0.001, p = 0.034, p = 0.020, and p < 0.001 respectively). The rate of fetal anomaly (2.83%) and admission to the neonatal care unit (2.37%) was lower in the group using Vitamin D (p = 0.054, and p < 0.001, respectively).

**Table 3:** Differences maternal and fetal outcomes between groups that received and did not receive vitamin D

Variables		Yes	No	p^2^
		n = 1,098^1^	n = 1,768^1^	
Age		26.09 ± 5.71	26.29 ± 6.02	0.37
Education Level, *n (%)*				0.2
	Uneducated	220.00 (20.04%)	318.00 (17.99%)	
	Primary Education	649.00 (59.11%)	1,103.00 (62.39%)	
	High School	229.00 (20.86%)	347.00 (19.63%)	
Economic Status, *n (%)*				**0.009**
	High	159.00 (14.48%)	229.00 (12.96%)	
	Intermediate	494.00 (44.99%)	719.00 (40.69%)	
	Lower	445.00 (40.53%)	819.00 (46.35%)	
Number of antenatal Follow up		4.60 ± 1.21	2.66 ± 1.43	**< 0.001**
Gestational Age at Birth		38.9 ± 1.82	38.56 ± 2.15	**< 0.001**
Birth Weight		3,195.46 ± 484.08	3,123.62 ± 538.65	**< 0.001**
Hb (g/dl)		12.23 ± 1.29	11.12 ± 1.53	**< 0.001**
Htc (%)		36.79 ± 3.98	34.35 ± 3.99	**< 0.001**
Iron (Fe) Supplement		1,087.00 (99.00%)	1,222.00 (69.12%)	**< 0.001**
Delivery Type				0.13
	Cesarean Section	399.00 (36.34%)	692.00 (39.14%)	
	Vaginal Delivery	699.00 (63.66%)	1,076.00 (60.86%)	
Primary C-section Prevalence		60.00 (5.46%)	103.00 (5.83%)	0.68
Preterm Labor		63.00 (5.74%)	164.00 (9.28%)	**< 0.001**
	Birth weight 1500–2500 (g)	48.00 (28.40%)	121.00 (71.60%)	**< 0.001**
	Birth weight < 1500 (g)	32.00 (2.91%)	90.00 (5.08%)	**0.005**
	Macrosomia (≥ 4000 g)	36.00 (3.28%)	45.00 (2.55%)	0.25
Hypertensive disorders of pregnancy		7.00 (0.64%)	28.00 (1.58%)	0.054
GDM		10.00 (0.91%)	21.00 (1.19%)	**0.034**
Postpartum hemorrhage		20.00 (1.82%)	52.00 (2.94%)	**0.02**
PPROM		8.00 (0.73%)	11.00 (0.62%)	0.9
Blood transfusion		22.00 (2.00%)	158.00 (8.94%)	**< 0.001**
Length of hospitalization, day		1.36 ± 0.83	1.52 ± 1.18	**< 0.001**
APGAR Scors				
	1.Minutes	7.09 ± 0.58	7.04 ± 0.75	0.95
	5. Minutes	8.98 ± 0.44	8.89 ± 0.67	**0.011**
NICU admission		26.00 (2.37%)	110.00 (6.22%)	**< 0.001**
Congenital fetal anomaly		50.00 (2.83%)	39.00 (3.55%)	0.054

Welch Two Sample t-test; Pearson’s Chi-squared test; Wilcoxon rank sum test; Fisher’s exact test, SD: Standard deviation, n: number, %: percent, CPD: cephalopelvic dispropotion, GDM: Gestational diabetes mellitus, PPROM: preterm premature rupture of membrane, NICU: Neonatal Intensive Care Unit

## Discussion

The Syrian civil war caused millions of Syrians to migrate to different countries and continue their lives in foreign geographies. In studies conducted with immigrant women in different countries, it has been reported that antenatal complications such as premature birth, low birth weight, increased frequency of cesarean section, bleeding during birth and increase in puerperal infections are more common. Maternal and fetal health can be affected by many variable factors under normal circumstances. The conditions of war and immigration may impose additional adverse effects on maternal and fetal health. Inadequate antenatal care, irregular nutrition, and insufficient vitamin intake are among the circumstances that can contribute to these negative effects. Konya is one of the provinces with the highest number of Syrian immigrants in Turkey. In our study, we examined the birth and newborn outcomes of Syrian refugee women, who had received the same service as the host population in a tertiary care center in terms of age groups and Vitamin D use.Previous studies have shown that refugee women encounter problems such as transportation difficulties, cost problems, safety concerns, and inadequate service while trying to reach prenatal health services [22, 23]. In Turkey, Syrians, like Turkish citizens, have been granted “temporary shelter status”, which gives them access to medical treatment and support free of charge, including prescription drugs.

In a study conducted by the United Nations High Commission for Refugees in Lebanon, it was shown that only 41% of pregnant Syrian women participated in four or more antenatal follow-ups [24]. This may be due to the complex health system in Lebanon and the provision of health services mostly by the private sector [7]. In our study, the average number of antenatal follow-ups of Syrian refugees was 2.9 under the age of 20, 3.6 over the age of 35, and 3.4 in general. The mean week of gestation at delivery was 38.6, mean hemogram and hematocrit concentrations were 11.55 g/dL and 35.28%, respectively. In addition, iron and Vitamin D supplement usage rates were 80.57% and 38.31%, respectively. In terms of newborn outcomes, in our study, the mean birth weight was 3.151 g, and the 1st and 5th minute APGAR scores were 7.06 and 8.92, respectively. Although APGAR scores are used as a physiological indicator of how well the newborn adapts to the external environment [25], many factors such as gestational age, low birth weight, congenital anomaly, trauma, and infection can affect the scores [26]. Iron deficiency and low hemoglobin levels can lead to low-grade chronic hypoxia, increasing the risks of LBW and preterm birth [27, 28]. Benage et al. showed that iron tablets, nutrients, and a diet rich in folic acid improved the overall health and thus pregnancy outcomes of Syrian refugee women [22]. In this study, Hb and Htc results did not indicate severe iron deficiency. Additionally, rates of iron supplementation are similar to those for pregnant Turkish women [29]. Again, in our study, the premature birth rate (7.92%) was lower, unlike in many studies in the literature [5, 30, 31]. In some studies, conducted in Turkey, average Hb concentrations (10.7 g/dl, 10.8 g/dl, respectively) and preterm birth rates (17.6% and 21.5%, respectively) in Syrian immigrant pregnant women were found to be significantly higher than those in native pregnant women [30, 31]. On the other hand, in another study conducted in Turkey, although the premature birth rate in Syrian immigrants was reported as 9.4%, no significant difference was observed when compared to local pregnant women [32]. In light of our findings, it seems consistent with the existing literature that Syrian pregnant women, who attended their antenatal follow-ups and did not experience severe iron deficiency anemia, gave birth to infants with normal birth timing and weight. This suggests that the free healthcare services in Turkey contribute to rapid adaptation and provide sufficient care in our region. Studies conducted in Turkey and Lebanon reported high caesarean section rates (35% and 36%) in Syrian immigrants, especially due to the high recurrent caesarean section rates [2, 33]. According to a study published in 2009, primary cesarean section rates in Syria vary between 10% and 15% [34]. In our study, the cesarean section rate of Syrian immigrant women was 38.07% and the primary cesarean section rate was 5.69%. The low rate of primary cesarean section confirms that the most common indication for cesarean section is a previous cesarean section. Our recurrent cesarean section rates may have been found to be high because we are a reference hospital. High fertility rates among Syrian refugees and limited access to and use of prenatal care can lead to undetected pregnancy complications, often resulting in emergency cesarean Sect. [7].

In this study, the mean age of Syrian women was 26, with 10.8% adolescents (< 20) and 10% older (> 35). While the adolescent birth rate is reported as 14.1% [35] in Syria, the WHO reports the adolescent pregnancy rate as 10.8% worldwide [10]. In another study conducted in Turkey, the adolescent pregnancy rate in Syrian women was found to be 10.8%, similar to our study [32]. In our study, in accordance with the literature, lower education and socioeconomic status and a shorter last pregnancy interval were observed in the adolescent age group [11, 36]. Many studies have found that adolescent pregnancies are dangerous because they can cause poor obstetric and neonatal outcomes [31, 37]. In this study, obstetric complications such as gestational hypertension, GDM, postpartum hemorrhage and the need for blood transfusions were found to be lower in adolescent pregnant women compared to levels recorded for other age groups, contrary to the literature. In addition, while PPROM and preterm birth rates were similar in all age groups, cesarean section rates were found to be significantly lower in the adolescent group. In line with our results, it has been shown in different studies that obstetric complications are fewer in adolescents [38] and that cesarean delivery rates are lower than in those in adults [39]. On the other hand, in a recent study, the risk of preterm birth and preeclampsia was found to be higher especially in obese adolescent pregnancies under 15 years of age [40]. In addition, in our study, it was observed that obstetric complications were higher especially in those of advanced age compared to other age groups. Many studies have associated advanced maternal age with adverse obstetric outcomes, such as GDM, placenta previa, cesarean delivery, prolonged hospitalization, and PPROM [36]. Our results may indicate that adolescent age alone cannot explain poor obstetric outcomes and advanced maternal age may be a greater risk factor.

In recent years, studies have focused on Vitamin D deficiency as a common problem of mothers and babies, and in this context, the definition of perinatal Vitamin D deficiency has gained importance. Maternal Vitamin D deficiency may be associated with problems such as eclampsia/preeclampsia, low birth weight/premature birth, as well as neonatal hypocalcemia and infantile rickets, and it is emphasized that its effects on the fetus may last a lifetime [41, 42]. The relationship between Vitamin D and anemia has been studied recently, demonstrating the potential roles of Vitamin D in iron homeostasis and erythropoiesis [43]. In our study, we found significant maternal and perinatal outcomes in the group that received and did not take Vitamin D supplements. Hb and Htc levels were significantly higher in the group that received Vitamin D supplementation compared to those in the group that did not receive Vitamin D supplementation [p < 0.001]. In relation to these hematological markers, postpartum blood transfusion requirements were significantly lower in the group receiving Vitamin D [2.00%, 8.94%, and p < 0.001, respectively]. In addition to this information, we found that the need for preterm birth and neonatal intensive care was lower in the group receiving Vitamin D: 5.74% versus 9.28% [p < 0.001] for preterm delivery, and 2.37% versus 6.22% (p < 0.001) for neonatal intensive care. In addition, the rate of low birth weight infants (< 2500 g) was significantly lower in the group receiving Vitamin D. In a study by Akdulum et al. in which they examined the perinatal outcomes of 290 patients with Vitamin D deficiency, they could not show a relationship between Vitamin D and GDM, SGA, and preeclampsia [44]. However, Yuan-Hua Chen et al. revealed that maternal Vitamin D deficiency increases the risks of SGA and LBW babies not only in the early stages, but also in the middle and late pregnancy stages [45]. As in studies reporting that Vitamin D deficiency is associated with GDM [46], we found a lower rate of GDM in the group receiving Vitamin D in our study (0.91% vs. 1.19%. P = 0.034). Although the positive effects of Vitamin D supplementation on maternal and fetal health in our study are consistent with the literature, it should not be forgotten that this process is a multifactorial process for the pregnant woman and the fetus.

The strengths of the study are that it was conducted in a tertiary center and had a large sample group. Evaluation of adolescent and advanced age pregnancies is important in terms of explaining the differences in obstetric and fetal outcomes according to age groups. In addition, it is important to draw secondary conclusions that Vitamin D supplement intake can improve maternal and fetal outcomes. Apart from the strengths, there were some limiting factors in our study. First and most importantly, since we did not have a local pregnant control group, we were only able to evaluate the pregnancy and newborn outcomes of Syrian women by comparing them with different studies conducted on this subject in Turkey. Therefore, our findings may not coincide exactly with the actual results. Another limiting factor is that although we had laboratory results in which we could evaluate the intake of iron supplements, we evaluated Vitamin D intake only through retrospective data, since blood Vitamin D levels are not a parameter screened in routine pregnancy follow-up in Turkey. To achieve more definitive results, conducting studies with a control group and assessing Vitamin D levels would be beneficial. As a result, thanks to temporary shelter status in Turkey, Syrian refugees have access to medical treatment and support like Turkish citizens. To reduce pregnancy complications, it is important that refugee women are informed about pre-pregnancy care support, especially pregnancy spacing, family planning methods, number of visits and vitamin support. Although there is evidence that micronutrient deficiencies adversely affect maternal health and pregnancy outcome, no single micronutrient is thought to be responsible for adverse effects. Conducting controlled supplementation studies for all relevant micronutrients may not be easy. However, studies are needed to at least reveal potential mechanisms that may explain their association with good pregnancy outcomes. Recently, due to the increasing number of migrants, some countries have experienced poor perinatal and obstetric outcomes in migrants. With the help of these studies, each country can define its own migrant health strategy within the framework of its own health system.

## Data Availability

The datasets used and/or analysed during the current study are available from the corresponding author on reasonable request.
